# The role of motion and number of element locations in mirror symmetry perception

**DOI:** 10.1038/srep45679

**Published:** 2017-04-04

**Authors:** Rebecca J. Sharman, Elena Gheorghiu

**Affiliations:** 1 University of Stirling, Department of Psychology, Stirling, FK9 4LA, Scotland, United Kingdom

## Abstract

The human visual system has specialised mechanisms for encoding mirror-symmetry and for detecting symmetric motion-directions for objects that loom or recede from the observers. The contribution of motion to mirror-symmetry perception has never been investigated. Here we examine symmetry detection thresholds for stationary (static and dynamic flicker) and symmetrically moving patterns (inwards, outwards, random directions) with and without positional symmetry. We also measured motion detection and direction-discrimination thresholds for horizontal (left, right) and symmetrically moving patterns with and without positional symmetry. We found that symmetry detection thresholds were (a) significantly higher for static patterns, but there was no difference between the dynamic flicker and symmetrical motion conditions, and (b) higher than motion detection and direction-discrimination thresholds for horizontal or symmetrical motion, with or without positional symmetry. In addition, symmetrical motion was as easy to detect or discriminate as horizontal motion. We conclude that whilst symmetrical motion per se does not contribute to symmetry perception, limiting the lifetime of pattern elements does improve performance by increasing the number of element-locations as elements move from one location to the next. This may be explained by a temporal integration process in which weak, noisy symmetry signals are combined to produce a stronger signal.

Mirror symmetry (henceforth ‘symmetry’) is an image property where one half of a stimulus reflects the other about a vertical axis. Symmetry is found throughout natural and man-made scenes, and is a salient visual feature to which the human visual system is highly sensitive. Symmetry perception is even possible at isoluminance^1^, that is in stimuli defined solely by chromatic contrast. Psychophysical, computational and brain imaging studies have shown that symmetry perception plays an important role in perceptual organisation^2^, in particular in figure-ground segregation (i.e. symmetry is a property of the ‘figure’ rather than the ‘ground’)^3^,^4^,^5^ and object recognition^6^,^7^,^8^. Symmetry perception elicits a distinctive pattern of brain activity^9^, involving a large network of extrastriate visual areas such V3a, V7 and LOC^10^,^11^. Although several recent studies have examined the contribution of simple visual attributes, such as colour^12^,^13^,^14^ and stereoscopic depth^15^,^16^,^17^ to symmetry perception, little if any, is known about the role of *motion* in symmetry perception. In dynamic scenes, both motion direction and timing are likely to affect symmetry perception. In this communication, we provide new psychophysical evidence concerning the role of motion and the lifetime of pattern elements to symmetry perception.

It is generally believed that motion processing is mediated by the dorsal pathway and form processing by the ventral pathway^18^,^19^,^20^,^21^,^22^. However, there is increasing neurophysiological, anatomical and psychophysical evidence that motion and form processing interact. For example, anatomical studies in primates have found strong connectivity between the motion sensitive dorsal area MT and the form-sensitive ventral area V4^20^,^21^,^23^,^24^. Neurophysiological studies have also shown that neurons in ventral areas V4 and IT are sensitive to form defined by coherent motion of random dots^25^,^26^,^27^,^28^,^29^. The Gestalt principle of ‘common fate’ allows seemingly random dots to become recognisable as a pattern when they move coherently^30^. For example, a point light walker embedded in a random dot pattern is not detectable when static, but is instantly recognisable when the dots are moving coherently^31^.

Psychophysical evidence for an interaction between form and motion comes from studies that examine the effect of motion on contour detection in background noise^32^,^33^,^34^,^35^,^36^,^37^,^38^ and the selectivity of contour shape to motion direction^39^. Evidence also comes from studies investigating the effect of motion-streaks and static gratings on the motion after-effect^40^,^41^,^42^,^43^,^44^,^45^,^46^ and from biological motion perception using point-light walkers^47^,^48^. Treder and Meulenbroek^49^ demonstrated that the presence of positional symmetry affects structure-from-motion percept duration in motion reversal tasks. This demonstrates that structure-from-motion incorporates both motion and form cues and more specifically that positional symmetry can interact with motion to change percept duration. There are, however, no studies that directly examine the role of motion in symmetry processing.

Psychophysical and neurophysiological studies have shown that the human visual system has specialised motion detectors for objects that loom (move towards) and recede (move away) from the observer^50^. Regan and colleagues^50^ have shown that monocular (2D) motion in depth mechanisms are reliant on detecting symmetrical motion-directions and velocities, such as expansion (outwards) or contraction (inwards) motions. The visual system must, therefore, be sensitive to departures from symmetry in order to be able to use these mechanisms to extrapolate an object’s trajectory in space. This may suggest that inwards or outwards symmetrical motion could facilitate symmetry perception. In light of this, we investigate whether combining positional symmetry with symmetrical motion signals will facilitate the detection of symmetry and whether symmetrical motion may be easier to detect or discriminate than horizontal motion.

Investigations into motion perception often use stimuli consisting of limited-lifetime dots to avoid tracking of individual pattern elements^51^. In this type of stimuli each dot is assigned a different ‘starting age’ at stimulus onset and then relocated when the maximum lifetime duration has been reached. For symmetric stimuli, this means that pairs of dots are relocated asynchronously, giving a flickering or twinkling appearance (see Movie S1). Conversely, studies of symmetry perception generally employ static patterns in which the elements do not change over time^13^,^52^,^53^. It has been found, however, that rapid consecutive presentation of different symmetrical patterns improves orientation discriminations of the axis of symmetry^54^,^55^. Therefore, dynamically changing the position of the pattern elements over time (without coherent motion) by asynchronously relocating pairs of dots may also facilitate perception of symmetry. The extent to which lifetime of the pattern elements (in the absence of coherent motion) affects symmetry perception has never been investigated.

To determine the contribution of motion to symmetry perception we compared *symmetry detection thresholds* obtained with random-dot patterns containing either positional symmetry only (Fig. 1a) or both positional and motion symmetry (Fig. 1b–d). Dynamic versions of these ‘with-symmetry’ stimuli are shown in the Supplementary Information (see Movies S1, S2, S3 and S4). The positional symmetry only condition is actually two conditions: a ‘static’ condition in which a single static pattern was presented and a ‘dynamic flicker’ condition in which the dots had a limited lifetime and there was no coherent global or local motion. For the motion and positional symmetry conditions, dots had limited lifetimes and their global motion direction was inwards (Fig. 1b), outwards (Fig. 1c) or random (Fig. 1d) i.e. matched pairs moved symmetrically but there was no coherent pattern of global motion. Noise dots followed the same motion direction as signal dots, but lacked positional symmetry. In both ‘dynamic flicker’ and ‘motion and symmetry’ conditions the lifetime of elements was the same. To examine the extent to which lifetime of pattern elements by itself (i.e. without coherent global or local motion) affects symmetry perception we measured symmetry detection thresholds for three different dot-lifetimes. If reducing the elements’ lifetime decreases symmetry detection thresholds then this would suggest that it is the *increased number of element locations* that is driving the improved performance.

Symmetry detection was measured using a two-interval forced choice (2IFC) task. On each trial a target ‘with symmetry’ and a foil ‘without symmetry’ were presented in different intervals for 400 ms with an inter-stimulus interval (ISI) of 400 ms. The ‘without symmetry’ patterns contained only noise dots moving in the same direction as the signal dots, but without positional symmetry. Participants indicated the interval containing positional symmetry by pressing a key. If symmetrical motion directions directly contribute to the perception of positional symmetry (as suggested by Regan *et al*.^50^) then one would expect symmetry detection thresholds to be lower for the combined position and motion symmetry conditions (i.e. inwards and outwards) compared to the position-only symmetry conditions (i.e. static and dynamic flicker). However, if any symmetrical local-motion signal (in the absence of coherent global motion) can contribute to symmetry perception then thresholds will also be reduced in the ‘random’ condition. If only limiting the lifetime of pattern elements affects symmetry perception one would expect thresholds for the ‘static’ condition to be different from the other four conditions.

The visual system is sensitive to symmetrical motion directions^50^, but it remains to be determined to what extent motion-defined symmetry and positional symmetry interact in symmetry perception. To establish the contribution of symmetrical motion directions (inwards/outwards) to symmetry perception, we compared *symmetrical motion detection thresholds* for patterns containing symmetrical motion (inwards/outwards) with and without positional symmetry with those obtained with horizontal (left/right) motion directions (Fig. 2). Dynamic versions of these stimuli are shown in the Supplementary Information (see Movies S5,S6,S7,S8,S9,S10,S11). In these patterns, signal and noise dots were segregated by motion direction (i.e. signal dots move horizontally and noise dots move in random directions). Motion detection thresholds were measured using a 2IFC task. On each trial a ‘with coherent motion’ stimulus and a ‘without coherent motion’ stimulus were presented in different intervals for 400 ms with an ISI of 400 ms and participants had to indicate the interval containing symmetrical or horizontal motion by a key press. If positional symmetry contributes to perception of symmetrical motion one would expect thresholds to be lower for the conditions containing both positional and motion symmetry. Similarly, if symmetrical motion is processed differently to horizontal motion one would expect to see differences in the motion detection thresholds for those two conditions.

It has been suggested that coherent motion detection and motion-direction discrimination tasks result in different performance (i.e. different thresholds) which might reflect different neural processes^56^,^57^. Therefore, for comparison, we also measured *direction discrimination thresholds* for symmetrical (inwards/outwards) and horizontal (left/right) motion directions (Fig. 2). Motion-direction discrimination thresholds were measured using a two alternative forced choice (2AFC) task. On each trial coherently moving signal dots were presented within randomly moving noise dots for 400 ms and participants had to indicate the coherent motion direction. As with the coherent motion detection experiment, if symmetrical motion is processed differently to horizontal motion one would expect to see differences in motion discrimination thresholds for these conditions.

## Results

### Does symmetrical motion direction contribute to symmetry perception?

Figure 3 shows symmetry detection thresholds (% positional symmetry signal) for individual observers and the average across observers for the static, dynamic flicker, inwards, outwards and random motion direction conditions. A repeated measures one-way analysis of variance (ANOVA) was conducted on the individual observers’ data to examine whether symmetry detection thresholds differ across conditions. The p-values are those associated with the Greenhouse-Geisser correction for violation of sphericity. For clarity, the original degrees of freedom are reported. The ANOVA showed a significant main effect of stimulus condition, F(4,32) = 24.264, p = 0.001, η^2^ = 0.75. Bonferroni corrected post-hoc analysis showed (a) a significantly higher mean threshold for the static condition (79.98%) compared to the other conditions (i.e. dynamic flicker: t(8) = 6.748, p = 0.001; inwards: t(8) = 8.693, p = 0.001; outwards: (t(8) = 6.304, p = 0.02 and random: t(8) = 7.296, p = 0.001); (b) comparable mean thresholds between the dynamic flicker (66.95%) and inwards (67.34%), outwards (65.89%) and random conditions (60.26%) (all post-hoc tests were not significant at α = 0.05) and (c) slightly lower mean threshold for the symmetrical but random motion directions compared to the inwards condition (t(8) = 3.98, p = 0.041). The comparable performance between dynamic flicker and symmetrical motion conditions, indicates that symmetrical motion per se does not contribute to symmetry perception, and limiting the lifetime of pattern elements does improve performance. This will be examined in the next experiment.

### Does the duration of dot lifetime affect symmetry detection thresholds?

In order to examine the extent to which lifetime of pattern elements in the absence of coherent motion affect symmetry perception we measured symmetry detection thresholds for three different dot-lifetimes. We predict that if reducing the elements’ lifetime decreases symmetry detection thresholds, then it is the increased number of element locations that is driving the improved performance.

Figure 4 shows symmetry detection thresholds (% positional symmetry signal) for individual observers and the average across observers for three durations of dot-lifetime: 27 frames (317.65 ms), 18 frames (211.77 ms) and 9 frames (105.88 ms). For all three dot-lifetime conditions, the stimulus presentation duration was the same (400 ms). A repeated measures one-way ANOVA was conducted on the individual observers’ data to examine whether there was an effect of lifetime on symmetry detection thresholds. The p-values are those associated with the Greenhouse-Geisser correction for violation of sphericity. For clarity, the original degrees of freedom are reported. The ANOVA showed a significant main effect of elements’ lifetime condition, F(2,12) = 8.884, p = 0.007, η^2^ = 0.597. Bonferroni corrected post-hoc tests showed that mean symmetry detection thresholds were significantly lower for the 18 frames (57.92%) than the 27 frames (68.21%) condition (t(6) = 3.569, p = 0.035). However the 9 frames condition (65.09%) was not significantly different from either the 27 frames condition (t(6) = 1.635, p = 0.459) or the 18 frames condition (t(6) = −2.766, p = 0.098). This may suggest that symmetry perception is tuned to a particular range of temporal frequencies.

### Does positional or motion symmetry affect coherent motion detection thresholds?

The previous experiments demonstrate the symmetrical-motion directions do not contribute to positional symmetry detection thresholds. However, it may be that motion-defined symmetry and positional symmetry interact in *coherent symmetrical-motion detection*. To test this, we compared motion detection thresholds for patterns containing symmetrical motion (inwards/outwards) with and without positional symmetry. For completeness, we also measured motion detection thresholds for patterns containing horizontal (left/right) motion directions.

Figure 5 shows individual observer and the average across observers’ motion detection thresholds (% coherent motion) for positional and motion symmetry (inwards, outwards), symmetrical motion only (inwards, outwards) and horizontal motion (left, right) conditions. A repeated measures one-way ANOVA was conducted on the individual observers’ data. The p-values are those associated with the Greenhouse-Geisser correction for violation of sphericity. For clarity, the original degrees of freedom are reported. The analysis showed comparable motion detection thresholds across all conditions, with no statistically significant differences in motion coherence thresholds between any of the conditions (F(5,35) = 3.075, p = 0.055, η^2^ = 0.305). Bonferroni corrected post-hoc tests showed no significant differences in detection thresholds between the inwards and outwards symmetrical motion directions (with or without positional symmetry) and horizontal (left/right) motion directions.

### Does motion symmetry affect direction discrimination thresholds?

The previous experiment showed that positional symmetry does not improve coherent symmetrical-motion detection. This demonstrates that motion-defined symmetry and positional symmetry do not interact in coherent motion detection. However, it has been suggested that coherent motion detection and motion-direction discrimination tasks could be underpinned by different mechanisms^56^,^57^. Therefore, for comparison, we measured motion-direction discrimination thresholds.

Figure 6 shows individual observer and the average across observers’ motion-direction discrimination thresholds (% coherent motion) for symmetrical motion (inwards, outwards) and horizontal motion (left, right). The individual observers’ data were submitted to a repeated measures one-way ANOVA to examine whether motion direction-discrimination thresholds differ between the conditions. The p-values are those associated with the Greenhouse-Geisser correction for violation of sphericity. For clarity, the original degrees of freedom are reported. The ANOVA showed a significant main effect of condition, F(3,18) = 5.961, p = 0.015, η^2^ = 0.498. Bonferroni corrected post-hoc tests showed a higher mean threshold for outwards motion (23.95%) compared to leftwards (17.84%)(t(6) = −4.897, p = 0.016) and rightwards (13.99%)(t(6) = −4.930, p = 0.016) motion directions. However, there was no significant difference between inwards (19.27%) and outwards (23.95%) conditions (t(6) = −2.237, p = 0.4).

### Comparisons between experiments

Figure 7a shows a direct comparison of the results for the inwards (dark green bars) and outwards (light green bars) symmetrical motion and position condition obtained in the symmetry detection experiment and symmetrical motion only conditions in the coherent motion detection and direction discrimination experiments. One can see that on average, the symmetry detection thresholds (65.07%) were about twice as large as the coherent detection thresholds (30.42%) and even larger than the motion direction discrimination thresholds (24.53%). A repeated measures two-way ANOVA with factors experiment type (symmetry detection vs coherent motion detection vs motion-direction discrimination) and stimulus motion-direction condition (inwards vs outwards) was conducted on the individual observers’ data. There was a significant main effect of experiment type (F(2,12) = 154.176, p = 0.001, η^2^ = 0.963). Bonferroni corrected post-hoc analysis showed that all experiment types were different from each other: symmetry detection thresholds were significantly higher than motion detection thresholds (t(6) = 14.273, p = 0.001) and direction discrimination thresholds (t(6) = 18.181, p = 0.001) and, motion detection thresholds were significantly higher than direction discrimination thresholds (t(6) = −11.405, p = 0.002). There was no significant main effect of motion-direction condition (F(1,6) = 1.071, p = 0.341, η^2^ = 0.152). However, there was a significant interaction between experiment type and condition (F(2,12) = 5.246, p = 0.035, η^2^ = 0.466) which appears to be driven by the raised thresholds in the outwards motion-direction discrimination condition.

Figure 7b shows motion detection (light green bars) and direction discrimination (dark green bars) thresholds obtained with horizontal (left, right) and symmetrical (inwards, outwards) motion. On average, motion detection thresholds (29.03%) were about 1.5 times higher than direction discrimination thresholds (19.58%). A repeated measures two-way ANOVA with factors experiment type (coherent motion detection vs motion-direction discrimination) and stimulus condition (left vs right vs inwards vs outwards) was conducted on the individual observers’ data. There was a statistically significant main effect of experiment type (F(1,16) = 62.549, p = 0.001, η^2^ = 0.912) confirming that the motion direction discrimination thresholds were significantly lower than the coherent motion detection thresholds. The main effect of stimulus condition was also significant (F(3,18) = 6.951, p = 0.008, η^2^ = 0.537). Bonferroni corrected post-hoc analysis showed a significant difference between rightwards and outwards conditions (t(6) = −5.375, p = 0.012), but no other significant differences. There was no significant interaction between type of condition and experiment (F(3,18) = 3.552, p = 0.066, η^2^ = 0.372).

## Discussion

We examined the role of motion in mirror-symmetry perception using random-dot patterns containing either positional symmetry only or symmetrical motion, with and without position symmetry. Our results show that symmetry detection thresholds are (i) significantly higher for static patterns, but there was no difference between the dynamic flicker and symmetrical motion and position conditions, and (ii) higher than motion detection and direction-discrimination thresholds for horizontal or symmetrical motion (with or without positional symmetry). We also found that symmetrical motion (inwards, outwards) detection thresholds are not affected by positional symmetry, nor are they significantly different from horizontal (left, right) motion detection thresholds. In addition, in the absence of positional symmetry, motion direction discrimination thresholds for symmetrical and horizontal motion directions were found to be similar. However, the overall performance was better for motion-direction discrimination than for motion detection thresholds.

It may seem surprising that performance levels for static symmetric pattern require about 80% symmetry signal. However, this level of performance is similar to those obtained by previous studies^12^,^53^. Our results show that static patterns have a higher detection threshold than moving patterns, but only when one single pattern is presented throughout. If the elements of the static pattern have a limited lifetime (i.e. the dynamic flicker condition), the thresholds become comparable for dynamic flicker and moving patterns. Therefore, the difference in performance between static and moving conditions is driven by the limited lifetime of the elements in the motion conditions. Continuous motion greatly increases the number of location samples as elements move from one location to the next. Nonetheless, the location information from continuous symmetrical-motion does not seem to be retrievable in order to contribute to judgements of positional symmetry. The onset of elements at new locations (when new lifetimes begin) seem to be necessary. Thus, there is no evidence that symmetrical motion directions contribute to the perception of positional symmetry.

In the light of Regan *et al*.’s^50^ finding that the visual system has neural mechanisms sensitive to symmetrical moving edges, it is surprising that motion direction does not contribute to the perception of positional symmetry. It may be that these ‘looming’ and ‘receding’ detectors are specific to object motion. Positional symmetry has also been found to influence perceptual durations of different 3D interpretations of structure-from-motion stimuli^49^. Treder and Meulenbroek^49^ have suggested that motion direction signals from V5/MT are feed-forwarded to higher stages of symmetry processing (e.g. area LOC) where they are integrated with positional symmetry signals used for surface interpolation. However, an alternative explanation for Treder and Meulenbroek^49^ findings is that symmetry signals from area LOC are sent to motion-selective area V5/MT via feed-back connections.

Our results show that dynamic stimuli produce lower thresholds than single, static patterns. A similar advantage has been found with dynamic glass patterns^58^,^59^, the regularities of which have been connected to symmetry^60^. Similarly, rapid consecutive presentation of different symmetrical patterns has been found to produce more accurate discriminations of orientations of axis of symmetry^54^,^55^. Niimi and collegues^54^ reported that this dynamic advantage was due to a mechanism that combines multiple neural responses over time. In other words, weak symmetry signals could be combined together to elicit a relatively stronger response. Thus, it is likely that the improved symmetry detection with dynamic flicker patterns that we found in our experiment is due to a similar cumulative temporal process.

In order to understand how this cumulative process might work, one needs a model of symmetry perception that accounts for rapid presentation time. This precludes accounts that are exclusively reliant on pair-wise comparisons or pixel-by-pixel correlation between symmetric halves of the images^61^,^62^. Symmetry can be detected pre-attentively^3^ or with very short presentation times^55^ and this is difficult to reconcile with accounts that require a more detailed examination of the pattern^13^. It has been suggested that there may be two different mechanisms underlying symmetry detection: one based on a crude, rapid response with a bias towards perceiving symmetry (i.e. it is more likely to miscategorise an asymmetrical stimulus as symmetrical than vice versa) and the other based on a slower response which is more accurate and detail oriented^55^,^63^. If this is the case, then the dynamic stimuli in our study would only be detected by the first mechanism because, even though the overall presentation time is the same as for static stimuli, the patterns presented were constantly changing and no one pattern was presented for more than a few milliseconds. In the static condition, however, one single pattern was presented for the full duration allowing the time for the second more accurate mechanism to operate. It should be noted that although symmetry detection thresholds are lower for the dynamic stimuli this does not mean that the faster mechanism is the most accurate. The lower thresholds may reflect an inherent perceptual bias for symmetry detection (even in the absence of symmetry). This may have utility in the real world situations (e.g. breaking camouflage), however, it is still a bias and as such the higher thresholds may be a more accurate reflection of the amount of symmetry present.

Measures of neural activity can also offer some insights into how temporal integration of symmetry may work. Measurement of event related potentials (ERPs) using single, static patterns shows that symmetry perception elicits a sustained posterior negativity (SPN)^9^,^64^,^65^ which occurs over a relatively long time with a peak amplitude of ~300 ms^9^. Presenting a new symmetrical pattern before the response to the previous pattern returns to the baseline could lead to the successive SPN signals being combined. It might be the case that continuous rapid presentation of symmetrical patterns, could result in an SPN that increases in amplitude over time.

Varying the duration of element lifetimes does affect symmetry detection thresholds. We found that thresholds are reduced for lifetimes of 18 frames duration compared to 27, but not for durations of 9 frames compared to 27. The dynamic-stimulus advantage found by Niimi and colleagues^54^ showed a similar pattern with performance improvements when the symmetrical patterns were changed at a moderate pace (4.7–18.8 Hz), but not when they were changed more rapidly or more slowly. This could suggest that the temporal properties of symmetry perception are tuned to a particular range of temporal frequencies.

Our experiments indicate that symmetrical motion detection thresholds are not affected by positional symmetry and are comparable with horizontal motion detection thresholds. This suggests that positional symmetry of the elements is not being utilised and that thresholds are entirely determined by coherent motion. We also found no significant difference in motion-direction discrimination thresholds between horizontal and symmetrical motion patterns although there is a hint in the data that outwards motion is harder to discriminate than other directions (compare light green bars in Fig. 7b).

It may be expected that the human visual system would have a greater sensitivity for outwards (centrifugal) patterns due to observers’ predominant experience of moving forward through the world. However, studies have found that for foveal viewing there is a bias for inwards (centripetal) motion in optic flow patterns^66^,^67^. Although our stimuli are not typical optic flow patterns, they do share several of their properties and therefore, the same mechanisms may underlie the slightly elevated outwards thresholds. Altogether our findings that there are no differences in participants’ abilities to detect or discriminate symmetrical motion compared to horizontal motion are in keeping with previous findings that radial motion detection and discrimination thresholds are comparable to those for horizontal motion^68^.

Motion direction discrimination thresholds are significantly lower than motion detection thresholds. This may be due to differences in task demands, the motion direction discrimination task was a two-alternative forced choice task (2AFC), whereas the motion detection task was a two-interval forced choice task (2IFC) which could have led to greater ambiguity in the motion detection task, increasing thresholds. However, ideal observer models have shown that the efficiency of motion direction discrimination relative to motion detection declines systematically with speed over the 1–6 Hz range, with motion discrimination being better than motion detection at higher speeds and poorer than motion detection at lower speeds^57^. In our experiment the speed was 2.47° per second. Thus, motion detection thresholds were found to be lower than motion-direction discrimination, in keeping with previous findings from ideal observer models^57^.

In conclusion, we have found that symmetry detection is better with dynamic than static patterns due to limited element lifetime. This might be explained by a cumulative temporal integration process whereby the increased number of symmetrical dot locations increases the perceived amount of symmetry, in keeping with previous findings^54^,^55^. We showed that symmetrical motion-direction does not improve symmetry detection and positional symmetry has no effect on the perception of symmetrical motion. In addition, we found that symmetrical motion (inwards/outwards) is not more difficult to detect or discriminate than horizontal motion (left/right). A direct comparison between symmetry-detection and symmetrical motion detection showed that symmetry detection thresholds were about twice as large as the coherence detection thresholds and even larger than the motion direction discrimination thresholds.

## Methods

### Participants

Nine observers participated in the symmetry detection experiment: the two authors and seven observers who were naive with regard to the experimental aims. For the limited-lifetime experiment, there were seven participants (two participants, AF and AR were unavailable) while in the motion detection and motion direction discrimination, there were eight participants (one participant, CM was unavailable). All participants had normal or corrected-to-normal vision. Observers gave their written informed consent and were treated in accordance with the Declaration of Helsinki (2008, Version 6). All procedures were approved by the Department of Psychology Ethics Committee, University of Stirling, UK.

### Stimuli – generation and display

Stimuli were presented on a gamma-corrected 20-in ViewSonic Professional Series PF817 cathode ray tube (CRT) monitor (ViewSonic, Brea, CA, USA) with spatial resolution of 1024 × 768 and refresh rate of 85 Hz. A ViSaGe MKII stimulus generator (Cambridge Research Systems, Cambridge, UK) in Bits# mode was used to control contrast. All stimuli were presented in the centre of the monitor on a mid-grey background with average luminance of 47.2 cd/m^2^. Viewing distance was 52 cm. All stimuli were generated and all data were collected using PsychoPy^69^.

Stimuli were presented in a square window of 9.03° (320 pixels) in width and were comprised of 40 circular white dots (100% contrast) of 0.17° (6 pixels) diameter. The symmetrical dots were positioned randomly on the left side of the stimulus area and then mirrored about the vertical axis onto the right side. Noise dots were positioned randomly such that equal numbers appeared in each stimulus half. All dots were positioned a minimum of 0.77° (27 pixels) apart. This resulted in a stimulus dot density of 0.7 dots/degree^2^.

Limited-lifetime dots had a maximum life of 317.65 ms (27 frames) to avoid tracking of individual pattern elements, and after the maximum lifetime duration was reached they ‘died’ and were relocated. Starting ‘ages’ were randomly allocated so that different dot pairs reached their maximum lifetime and ‘died’ at different times. Each pair of symmetrical dots was relocated simultaneously in order to maintain the same level of symmetry throughout presentation but, with different pairs relocated asynchronously. This gave a flickering or twinkling appearance, hence this condition was named ‘dynamic flicker’ (see Movie S1). Moving dots had a speed of 2.4 degrees per second. Positionally-symmetric dot pairs always moved in symmetrical directions and always had synchronised life times.

**Figure 1:**
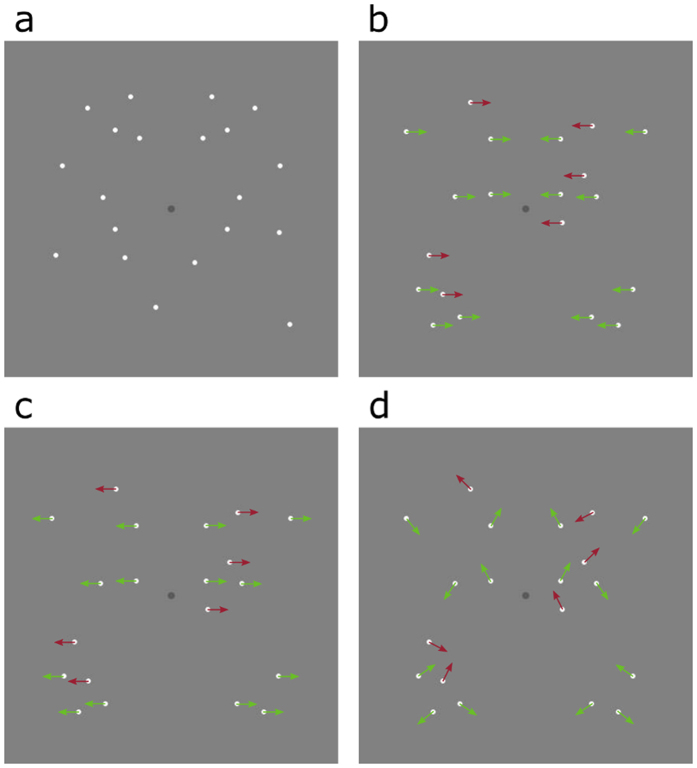
Example stimuli used in the symmetry detection experiment. Green and red arrows illustrate the symmetrical motion directions of positional symmetric and noise dots, respectively. (**a**) Static pattern containing positional symmetry only. (**b**–**d**) Symmetric patterns containing both positional and motion direction symmetry with (**b**) inwards, (**c**) outwards and (**d**) random (i.e. matched pairs moved symmetrically but there was no coherent pattern of global motion) directions. For illustrative purposes only, actual stimuli contained 40 dots.

**Figure 2:**
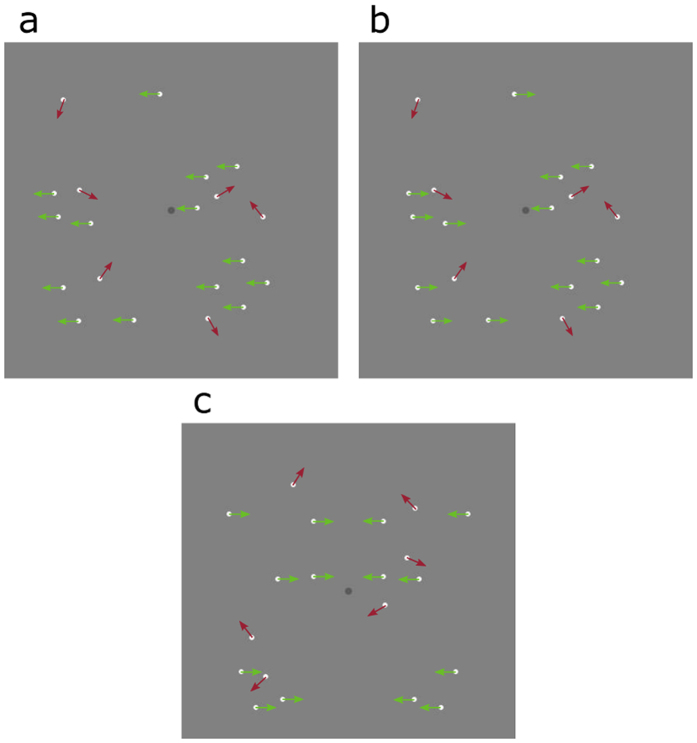
Example stimuli used in the coherent motion detection and motion direction discrimination experiments. Green and red arrows illustrate the motion directions of signal (i.e. coherently moving dots) and noise dots, respectively for (**a**) horizontal (leftward) motion and (**b**,**c**) symmetrical (inwards) motion for patterns without (**b**) and with (**c**) positional symmetry. For illustrative purposes only, actual stimuli contained 40 dots.

**Figure 3:**
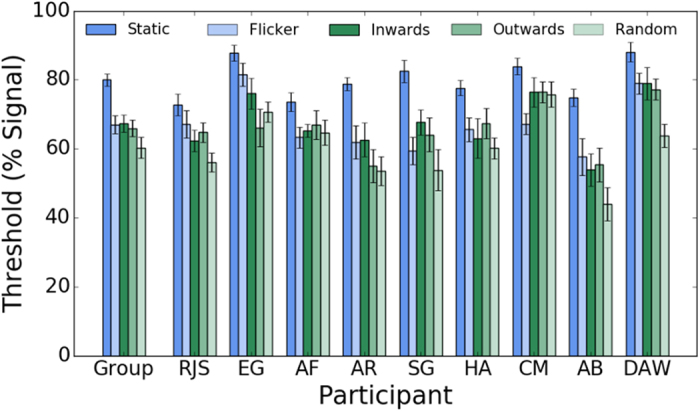
Average across observers and individual observer symmetry detection thresholds obtained with positional symmetry only (i.e. static (dark blue bars); dynamic flicker (light blue bars)) and, position and motion symmetry stimuli for the inward (dark green bars), outward (mid green bars) and random (light green bars) motion directions. Errors bars are ±1 SEM.

**Figure 4:**
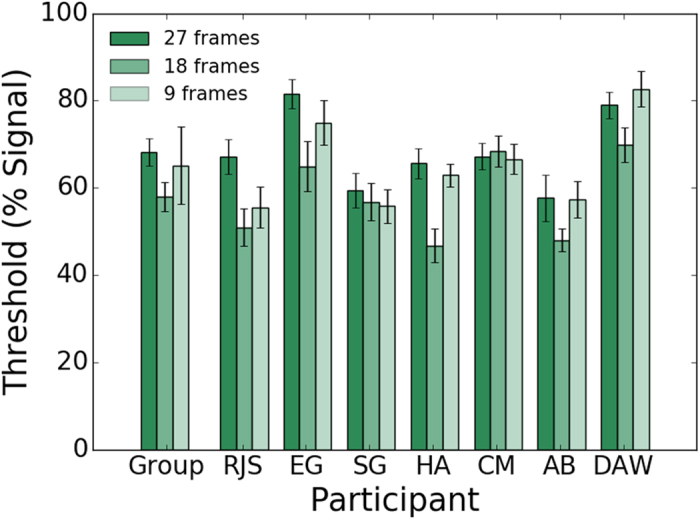
Average across observers and individual observer symmetry detection thresholds obtained in the dynamic flicker condition for three different durations of dot lifetimes (i.e. 27 frames (dark green bars); 18 frames (mid green bars) and 9 frames (light green bars). In all lifetime conditions, the stimulus presentation duration was the same (400 ms).Error bars are ± 1 SEM.

**Figure 5:**
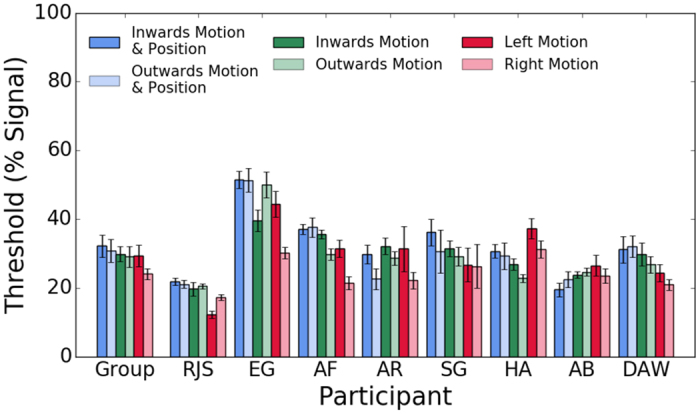
Average across observers and individual observer motion coherence detection thresholds obtained with position and motion symmetry stimuli (inwards (dark blue bars); outwards (light blue bars)), motion symmetry only stimuli (inwards (dark green bars); outwards (light green bars)) and horizontal motion stimuli (left (dark red bars); right (light red bars)). Error bars are ± 1 SEM.

**Figure 6:**
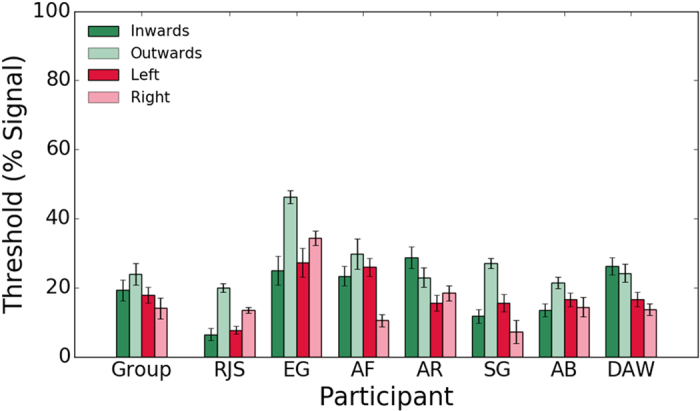
Average across observers and individual observer motion direction discrimination thresholds obtained with motion symmetry only stimuli (inwards (dark green bars); outwards (light green bars)) and horizontal motion stimuli (left (dark red bars); right (light red bars)). Error bars are ± 1 SEM.

**Figure 7:**
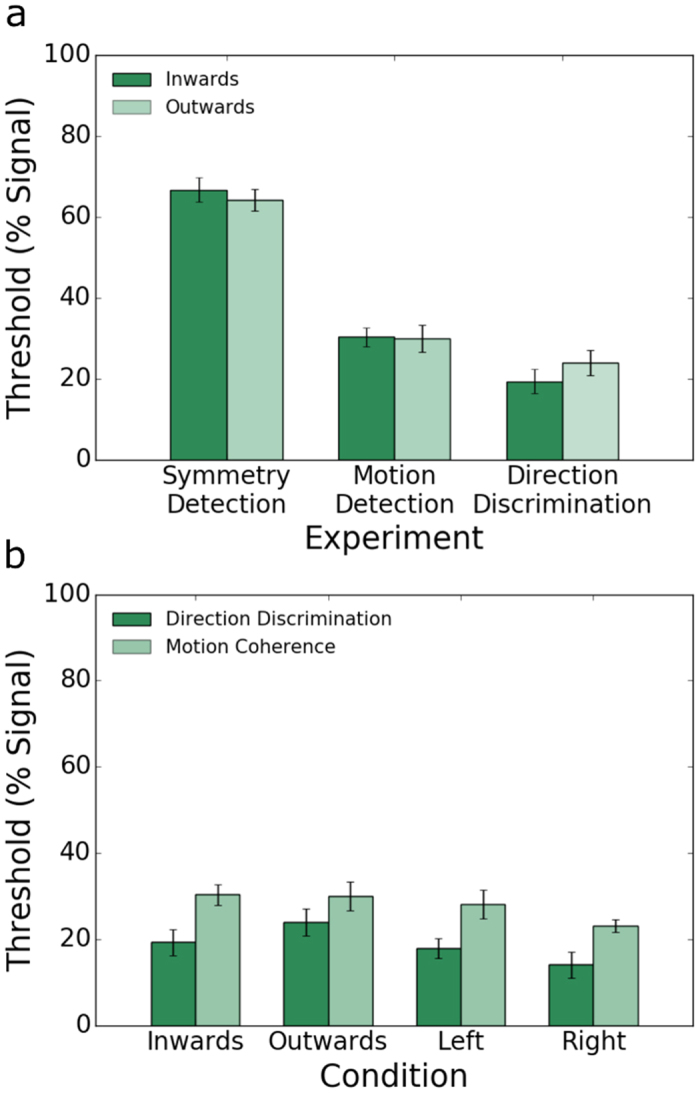
Average across observers for (**a**) symmetry detection, coherent motion detection and motion direction discrimination thresholds obtained with inwards (dark green bars) and outwards (light green bars) symmetrical motion and positional symmetry. (**b**) Motion direction discrimination thresholds (dark green bars) and motion coherence thresholds (light green bars) obtained with symmetrical motion (inwards, outwards) and horizontal motion (left, right). Error bars are ± 1 SEM.

For the symmetry detection experiment we used five ‘with symmetry’ stimulus conditions: 1) ‘static’ in which a single static pattern was presented for the entire trial (Fig. 1a); 2) ‘dynamic flicker’ in which the dots had a limited lifetimes of 317.65 ms as described above; 3) ‘inwards’ – the dots moved horizontally towards the axis of symmetry and had a limited lifetime of 317.65 ms (Fig. 1b); 4) ‘outwards’ – the dots moved horizontally away from the axis of symmetry and had a limited lifetime of 317.65 ms (Fig. 1c); 5) ‘random’ – corresponding positionally-symmetric dots moved in symmetrical directions, but with different pairs having randomly allocated directions and a limited lifetime of 317.65 ms (Fig. 1d). In all conditions, stimulus duration was 400 ms. Noise dots moved in the same directions as signal dots, but did not have positional symmetry. We also conducted an experiment with three different lifetime durations for the dynamic flicker condition only: 317.65 ms (27 frames), 211.77 ms (18 frames) and 105.88 ms (9 frames).

For the motion detection experiment we used six stimulus conditions: horizontal (left/right) motion (Fig. 2a), symmetrical motion without positional symmetry (inwards/outwards, Fig. 2b) and symmetrical motion with positional symmetry (inwards/outwards, Fig. 2c). For the direction discrimination experiment we used the same horizontal motion and symmetrical motion without positional symmetry conditions described above. Noise dots moved in random directions and had no correlated motion. Thus, signal and noise dots were segregated by motion direction.

### Procedure – symmetry detection thresholds

A two-interval forced choice (2IFC) design was employed to measure symmetry detection thresholds. In each trial a stimulus corresponding to one of the five conditions (i.e. static, dynamic, inwards, outwards and random) was randomly presented in one of the two intervals while the other interval (i.e. the foil or null interval) contained a stimulus consisting of randomly positioned dots. Each stimulus (i.e. foil and target) was presented for 400 ms with an inter-stimulus interval (ISI) of 400 ms. The task for the subject was to indicate by a key press which interval contained the positionally symmetric stimulus. The presentation order of the foil and target was randomised.

In each condition we varied the number of positionally symmetric dots and measured the minimum number of symmetric dots required for the participant to perceive the pattern as symmetrical (i.e. the symmetry detection threshold). Thresholds were measured using a one-up, three-down staircase procedure. The staircases controlled the number of positionally symmetric dots in the target patterns. In each run two staircases were interleaved: one starting with the target at 100% positional symmetry and the other starting with 0% positional symmetry. The staircases were designed to converge at the 83% threshold and were terminated after 75 trials. Participants were allowed as many practice runs as required to become familiar with the task. Each participant collected a minimum of ten staircases for each condition (750 trials). The mean of the last six reversals of each of the staircases was calculated for each condition and each participant. For participant HA one run (two staircases) had to be excluded from the inward condition because neither of the staircases converged. In addition to the reported analyses, data were reanalysed for all experiments with the authors data (EG and RJS) removed, see Supplementary Material (Appendix A) for details.

### Procedure – motion detection thresholds

A 2IFC design was employed to measure motion detection thresholds. Participants were presented with two intervals, one containing the foil and the other containing the target (i.e. one of the six conditions: left/right motion, inwards/outwards motion with and without positional symmetry). Each stimulus was presented for 400 ms separated by an ISI of 400 ms. In the horizontal motion condition participants were asked to indicate by a key press which interval contained horizontal motion and in the symmetrical conditions they were asked which interval contained symmetry. The presentation order of foil and target was randomised.

In each condition we measured the minimum number of coherently moving dots necessary for horizontal or symmetrical motion to be detected. This was done in order to determine whether there is a difference in the detection thresholds for patterns containing both symmetrical motion and positional symmetry and patterns containing symmetrical motion alone. Further, we examined whether either of these conditions was different from detection of horizontal motion patterns.

Staircases controlled the number of coherently moving dots in the target stimuli. In each run four staircases were interleaved, two for each motion direction with one starting with the target at 100% signal and the other starting at 0% signal. Runs for the horizontal and symmetrical motion conditions were alternated. One participant (HA) was unable to perform above chance in the horizontal motion condition and their data from both conditions were excluded from the analysis.

### Procedure – motion-direction discrimination thresholds

A two-alternative forced choice (2AFC) design was employed. Participants were presented with one stimulus containing either left/right motion or inwards/outwards motion without positional symmetry. In the horizontal motion condition, participants were asked to indicate by pressing a key whether the dots were moving to the left or right. For the symmetrical condition they were asked to indicate by pressing a key whether the dots were moving inwards or outwards. Each stimulus was presented for 400 ms and for each condition, the presentation order of the two possible motion directions (i.e. left vs right or inwards vs outwards) was randomised. For each condition we measured the minimum number of coherently moving dots necessary for motion direction to be discriminated. Staircases controlled the number of coherently moving dots in the target images. In each run four staircases were interleaved, two for each motion direction with one starting at 100% signal and the other starting at 0% signal. Runs for the horizontal and symmetrical motion conditions were alternated.

## Additional Information

**How to cite this article**: Sharman, R. J. and Gheorghiu, E. The role of motion and number of element locations in mirror symmetry perception. *Sci. Rep.* **7**, 45679; doi: 10.1038/srep45679 (2017).

**Publisher's note:** Springer Nature remains neutral with regard to jurisdictional claims in published maps and institutional affiliations.

## Supplementary Material

Supplementary Movie S1

Supplementary Movie S2

Supplementary Movie S3

Supplementary Movie S4

Supplementary Movie S5

Supplementary Movie S6

Supplementary Movie S7

Supplementary Movie S8

Supplementary Movie S9

Supplementary Movie S10

Supplementary Appendix A
