# Supplementary figures and images for: The role of motion and number of element locations in mirror symmetry perception
Source: Sci Rep. 2017 Apr 4;7:45679. doi: 10.1038/srep45679 (PMC5379492; doi:10.1038/srep45679)

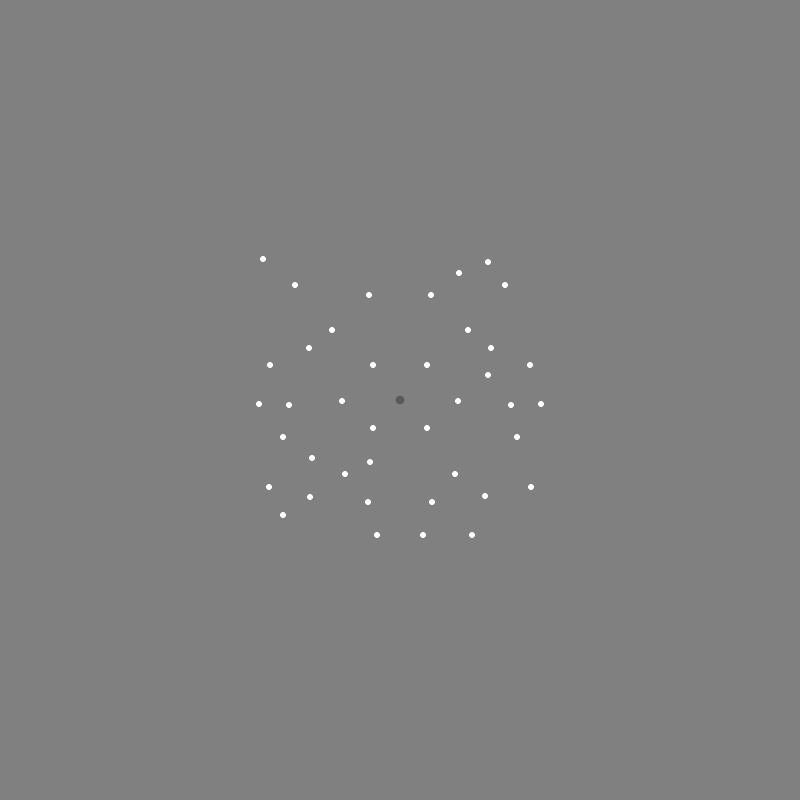

Supplement: Supplementary Movie S1 [file srep45679-s1.gif]

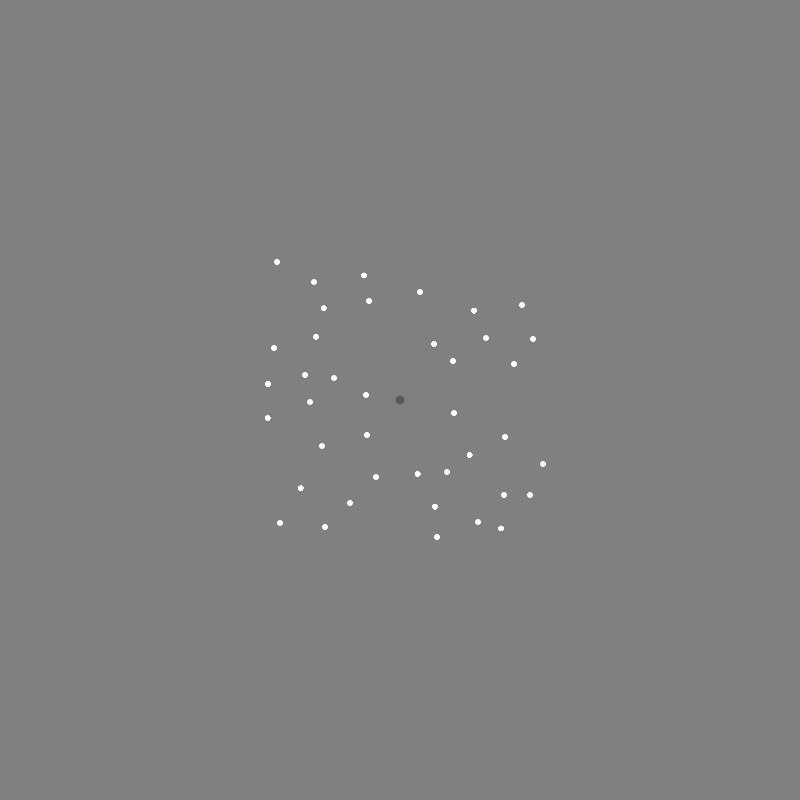

Supplement: Supplementary Movie S2 [file srep45679-s2.gif]

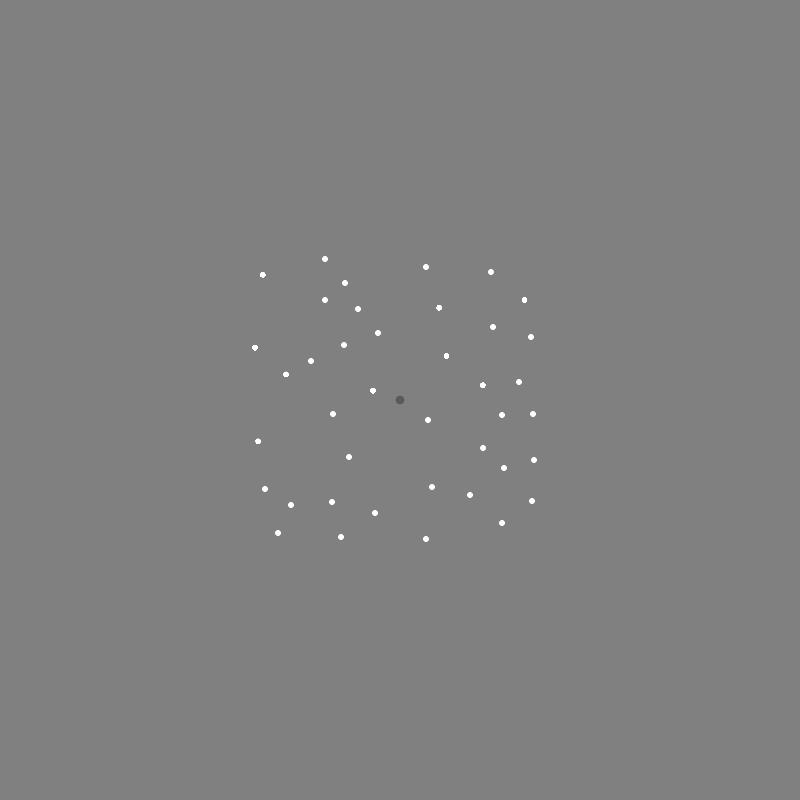

Supplement: Supplementary Movie S3 [file srep45679-s3.gif]

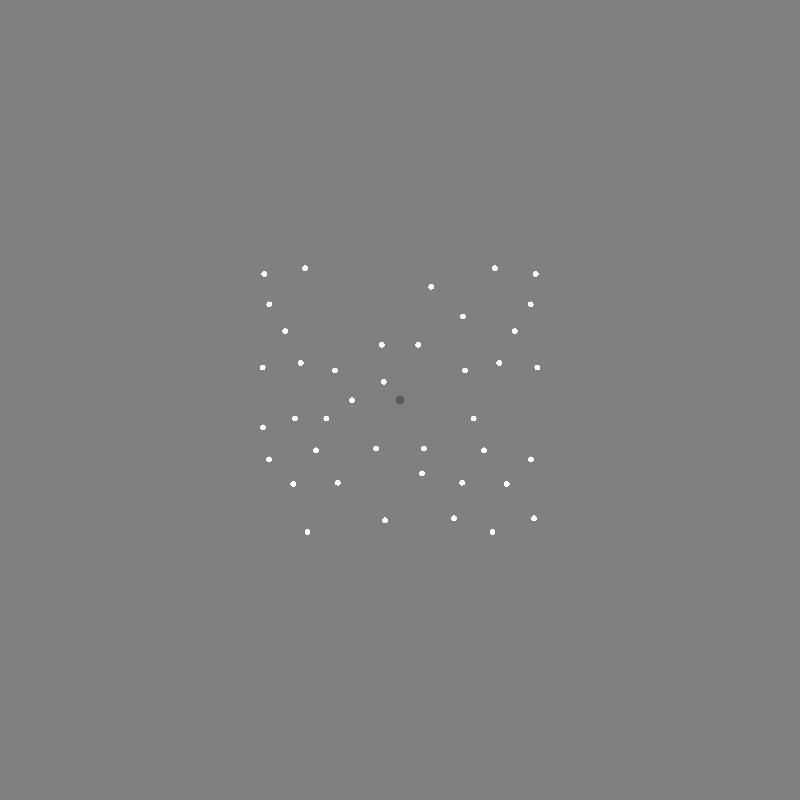

Supplement: Supplementary Movie S4 [file srep45679-s4.gif]

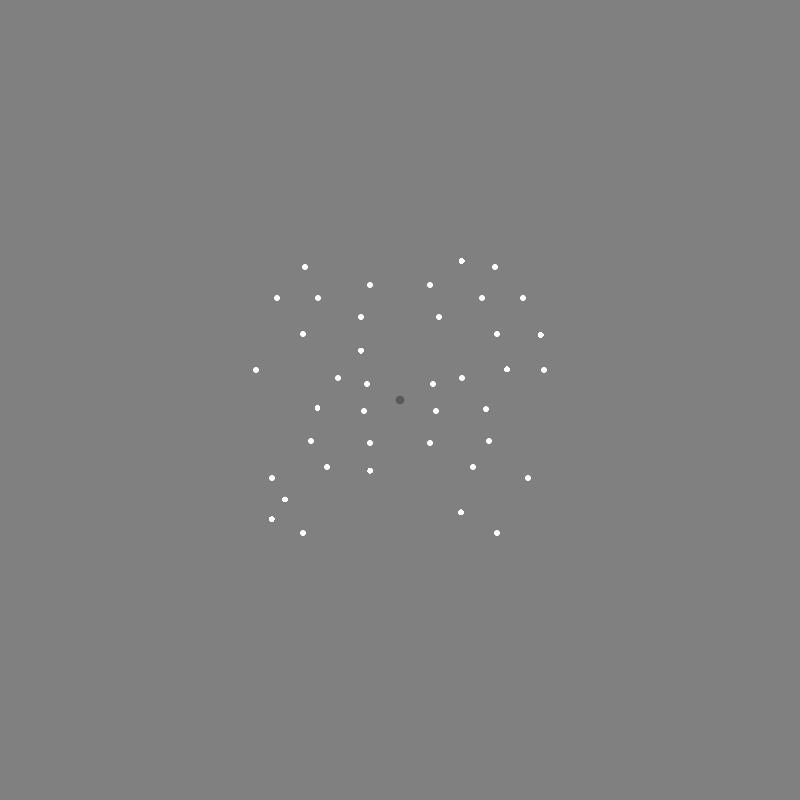

Supplement: Supplementary Movie S5 [file srep45679-s5.gif]

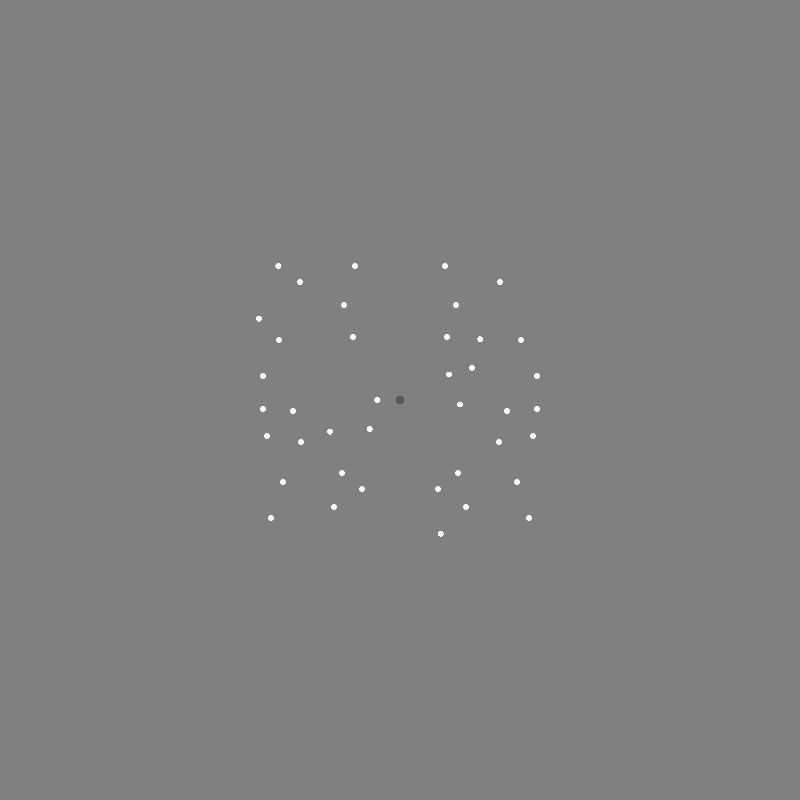

Supplement: Supplementary Movie S6 [file srep45679-s6.gif]

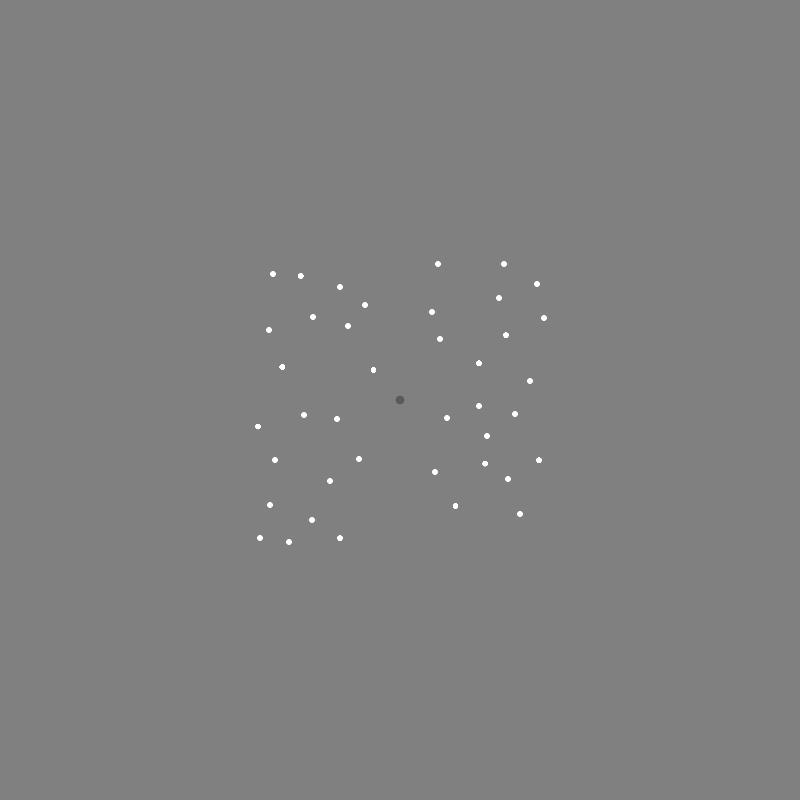

Supplement: Supplementary Movie S9 [file srep45679-s9.gif]

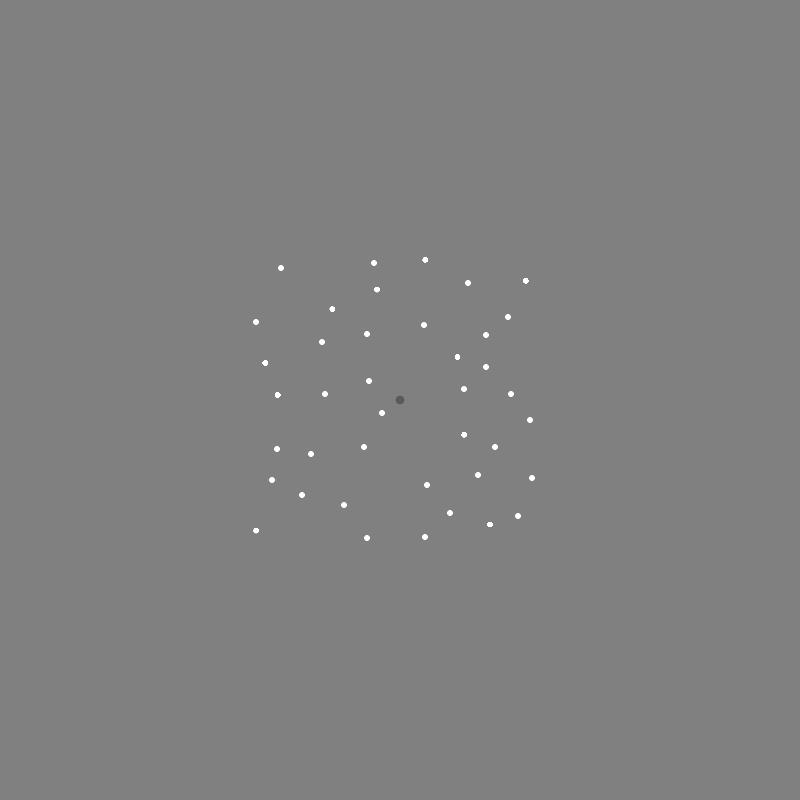

Supplement: Supplementary Movie S10 [file srep45679-s10.gif]
