# Supplementary material for: The role of motion and number of element locations in mirror symmetry perception
Source: Sci Rep. 2017 Apr 4;7:45679. doi: 10.1038/srep45679 (PMC5379492; doi:10.1038/srep45679)
Supplement: Supplementary Appendix A [file srep45679-s11.doc]

# Supplementary Information

# The role of motion and element lifetime in mirror symmetry perception

Rebecca J. Sharman^1^ & Elena Gheorghiu^1^

***^1^*** *University of Stirling, Department of Psychology, Stirling, FK9 4LA, Scotland, United Kingdom*

Corresponding author: [rebecca.sharman@stir.ac.uk](mailto:rebecca.sharman@stir.ac.uk)

## Appendix A – Statistical analysis results with authors’ data (participants EG and RJS) removed.

For all analyses, the p-values are those associated with the Greenhouse-Geisser correction for violation of sphericity. For clarity, the original degrees of freedom are reported.

#### Does symmetrical motion direction contribute to symmetry perception?

A repeated measures one-way analysis of variance (ANOVA) was conducted on the individual observers’ data to examine whether symmetry detection thresholds differ across conditions. The ANOVA showed a significant main effect of stimulus condition, F(4,24) = 21.238, p = 0.001, η^2^ = 0.78. Bonferroni corrected post-hoc analyses showed (a) a significantly higher mean threshold for the static condition compared to the other conditions (i.e. dynamic flicker: t(6) = 8.259, p = 0.002; inwards: t(6) = 7.005, p = 0.004; outwards: (t(6) = 5.517, p = 0.015 and random: t(6) = 5.899, p = 0.011); (b) comparable mean thresholds between the dynamic flicker and the inwards, outwards and random conditions (all post-hoc test were not significant at α =0.05).

#### Does the duration of dot lifetime affect symmetry detection thresholds?

A repeated measures one-way ANOVA was conducted on the individual observers’ data to examine whether there was an effect of lifetime on symmetry detection thresholds. The ANOVA showed a significant main effect of stimulus condition, F(2,8) = 3.964, p = 0.103, η^2^ = 0.498. Bonferroni corrected post-hoc tests showed no significant differences.

#### Does positional or motion symmetry affect coherent motion detection thresholds?

A repeated measures one-way ANOVA was conducted on the individual observers’ data. The analysis showed comparable motion detection thresholds across all conditions, with no statistically significant differences in motion coherence thresholds between any of the conditions (F(5,25) = 2.108, p = 0.161, η^2^ = 0.297). Bonferroni corrected post-hoc tests showed no significant differences in detection thresholds between the inwards and outwards symmetrical motion directions (with or without positional symmetry) and horizontal (left/right) motion directions.

#### Does motion symmetry affect direction discrimination thresholds?

The individual observers’ data were submitted to a repeated measures one-way ANOVA to examine whether motion direction-discrimination thresholds differ between the conditions. The ANOVA showed a significant main effect of condition, F(3,12) = 5.683, p = 0.035, η^2^ = 0.587. Bonferroni corrected post-hoc tests showed a higher mean threshold for outwards motion compared to leftwards (t(4) = -5.406, p = 0.034).

#### Comparisons between experiments

A repeated measures two-way ANOVA with factors experiment type (symmetry detection vs coherent motion detection vs motion-direction discrimination) and stimulus condition (inwards vs outwards) was conducted on the individual observers’ data. There was a significant main effect of experiment type (F(2,8) = 107.178, p = 0.001, η^2^ = 0.964). Bonferroni corrected post-hoc analyses show that all experiment types were different from each other: symmetry detection thresholds were significantly higher than motion detection thresholds (t(4) = 8.199, p = 0.002) and direction discrimination thresholds (t(4) = 11.610, p = 0.001) and, motion detection thresholds were significantly higher than direction discrimination thresholds (t(4) = -7.802, p = 0.028). There was no significant main effect of condition (F(1,4) = 0.004, p = 0.95, η^2^ = 0.001) and no significant interaction between experiment type and condition (F(2,8) = 3.279, p = 0.117, η^2^ = 0.45).

A repeated measures two-way ANOVA with factors experiment type (coherent motion detection vs motion-direction discrimination) and stimulus condition (left vs right vs inwards vs outwards) was conducted on the individual observers’ data. There was a statistically significant main effect of experiment type (F(1,4) = 38.943, p = 0.003, η^2^ = 0.907) confirming that the motion direction discrimination thresholds were significantly lower than the coherent motion detection thresholds. The main effect of stimulus condition was also significant (F(3,12) = 8.030, p = 0.008, η^2^ = 0.668). Bonferroni corrected post-hoc analysis showed a marginally significant difference between rightwards and outwards conditions (t(4) = -4.324, p = 0.052), but no other significant differences. There was no significant interaction between type of condition and experiment (F(3,12) = 3.053, p = 0.066, η^2^ = 0.433).

## Video Legends

**Movie S1.** Example of the ‘dynamic flicker’ stimulus condition from the positional symmetry detection experiment with 75% positional symmetry.

**Movie S2.** Example of the ‘inwards’ stimulus condition from the positional symmetry detection experiment with 75% positional symmetry.

**Movie S3.** Example of the ‘outwards’ stimulus condition from the positional symmetry detection experiment with 75% positional symmetry.

**Movie S4.** Example of the ‘random’ stimulus condition from the positional symmetry detection experiment with 75% positional symmetry.

**Movie S5.** Example of the inwards motion with positional symmetry condition from the motion coherence experiment with 75% positional symmetry and 75% coherent motion.

**Movie S6.** Example of the outwards motion with positional symmetry condition from the motion coherence experiment with 75% positional symmetry and 75% coherent motion.

**Movie S7.** Example of the inwards motion without positional symmetry condition from the motion coherence and direction discrimination experiments with 75% coherent motion.

**Movie S8.** Example of the outwards motion without positional symmetry condition from the motion coherence and direction discrimination experiments with 75% coherent motion.

**Movie S9.** Example of the leftwards motion without positional symmetry condition from the motion coherence and direction discrimination experiments with 75% coherent motion.

**Movie S10.** Example of the rightwards motion without positional symmetry condition from the motion coherence and direction discrimination experiments with 75% coherent motion.
